# Mechanisms of Training-Related Change in Processing Speed: A Drift-Diffusion Model Approach

**DOI:** 10.5334/joc.310

**Published:** 2023-08-18

**Authors:** Alice Reinhartz, Tilo Strobach, Thomas Jacobsen, Claudia C. von Bastian

**Affiliations:** 1 Department of Psychology, Medical School Hamburg, Hamburg, Germany; 2 Experimental Psychology Unit, Helmut Schmidt University/University of the Federal Armed Forces Hamburg, Hamburg, Germany; 3 Department of Psychology, University of Sheffield, Sheffield, UK

**Keywords:** Processing Speed, Cognitive Training, Drift-Diffusion Model

## Abstract

Processing speed is a crucial ability that changes over the course of the lifespan. Training interventions on processing speed have shown promising effects and have been associated with improved cognitive functioning. While training-related changes in processing speed are often studied using reaction times (RTs) and error rates, these measures provide limited insight into the mechanisms underlying changes during training. The drift-diffusion model provides estimates of the cognitive processes underlying speeded decision tasks, such as the rate of evidence accumulation (drift rate), response strategies (boundary separation), as well as time for other processes such as stimulus encoding and motor response (non-decision time). In the current study, we analyzed existing data of an extensive multi-session training intervention (von Bastian & Oberauer, 2013) to disentangle changes in drift rate, boundary separation, and non-decision time during training of different speeded choice-RT tasks. During this training intervention, 30 participants performed 20 training sessions over the course of four weeks, completing three tasks each session: a face-matching, a pattern-matching, and a digit-matching task. Our results show that processing speed training increased drift rates throughout training. Boundary separation and non-decision time decreased mostly during the initial parts of training. This pattern of prolonged training-related changes in rate of evidence accumulation as well as early changes in response strategy and non-decision processes was observed across all three tasks. Future research should investigate how these training-related changes relate to improvements in cognitive functioning more broadly.

## Introduction

People constantly make decisions based on the perceptual information around them. These decisions can occur naturally and rapidly, such as recognizing a friend on the street and greeting them before they have passed by. Before the decision is made to utter the friend’s name, perceptual information of this friend’s face and appearance is processed quickly enough to retrieve and then call out their name on time. The speed of processing perceptually available information, such as a familiar face or other information from our environment, differs between individuals and these differences are related to a range of individual differences in other cognitive abilities (e.g., Kail & Salthouse, 1994), including fluid intelligence (Sheppard & Vernon, 2008) and working memory (Schmiedek et al., 2007). Like other fluid cognitive abilities (Bialystok et al., 2012), processing speed changes over the course of the lifespan: it increases from childhood to adulthood and then decreases again from young to old adulthood (e.g., Salthouse & Kail, 1983).

In recent years, cognitive training interventions have been studied as a way of enhancing cognitive abilities, such as processing speed, and counteracting their age-related decline (Edwards et al., 2017, 2018; Simons et al., 2016; von Bastian et al., Belleville, et al., 2022). Whereas training interventions have targeted different cognitive domains with overall mixed results, training processing speed has shown promising effects and has been associated with improved cognitive functioning, improved everyday life functioning and even delayed onset of dementia (Edwards et al., 2017, 2018). To design training interventions that consistently show such far-reaching effects, it is important to understand which mechanisms facilitate these effects and induce changes already during training (von Bastian, Belleville, et al., 2022). The current study investigated the mechanisms underlying training-related changes in processing speed by examining the changes in the components of reaction time (RT) distributions estimated with the drift-diffusion model, using existing data of a multi-session training intervention (von Bastian & Oberauer, 2013).

### Measuring Performance in Speeded Decision Tasks

A common finding during cognitive training is that people improve in the trained tasks (e.g., von Bastian, Belleville, et al., 2022; Simons et al., 2016). These improvements are referred to as training effects, while improvements in tasks different from the trained tasks are called transfer effects. Processing speed can be assessed with simple tasks that require people to perceptually locate, classify, compare or merely detect stimuli presented to them. In a two-choice RT task, for example, participants are presented with one or more stimuli and two response options to choose from, such as viewing a face and categorizing it as familiar or not. Often, the presentation time of the stimuli is limited and participants are asked to respond both quickly and accurately. Performance on these speeded tasks is then measured by RTs and error rates with decreases in mean RT and error rate used as indicators for training effects.

However, the use of RTs and error rates as performance measures for training-related improvements in processing speed tasks has several limitations. First, RT distributions are often skewed (e.g., Micceri, 1989). Therefore, commonly used metrics such as the mean do not capture the RT distribution well. Second, processing speed tasks are designed so that the task itself is easy and can be performed well by most people. Therefore, error rates typically show ceiling effects, resulting in limited variance and, thus, interpretability. Third, the theoretical interpretation of improvements in RTs and error rates is not informative with regards to the specific cognitive mechanisms affected by training. For example, RTs can be reduced not only by improving the efficiency in stimulus information processing, but also by enhancing the speed of motor responses and perceptual encoding (Pashler & Baylis, 1991; Strobach et al., 2013). Finally, changes in RT can indicate improved task performance but also reflect strategic changes in speed-accuracy trade-offs. Whereas slow RTs can be the result of a more cautious response strategy favoring accuracy over speed, fast RTs can reflect favoring speed over accuracy (e.g., Schouten & Bekker, 1967; Wood & Jennings, 1976). Overall, while improved RTs and error rates are often used to measure training effects, these measures provide only limited information about the mechanisms underlying training-related change (see also von Bastian, Reinhartz, et al., 2022).

### Estimating Decision Components with the Drift-Diffusion Model

**Figure 1:**
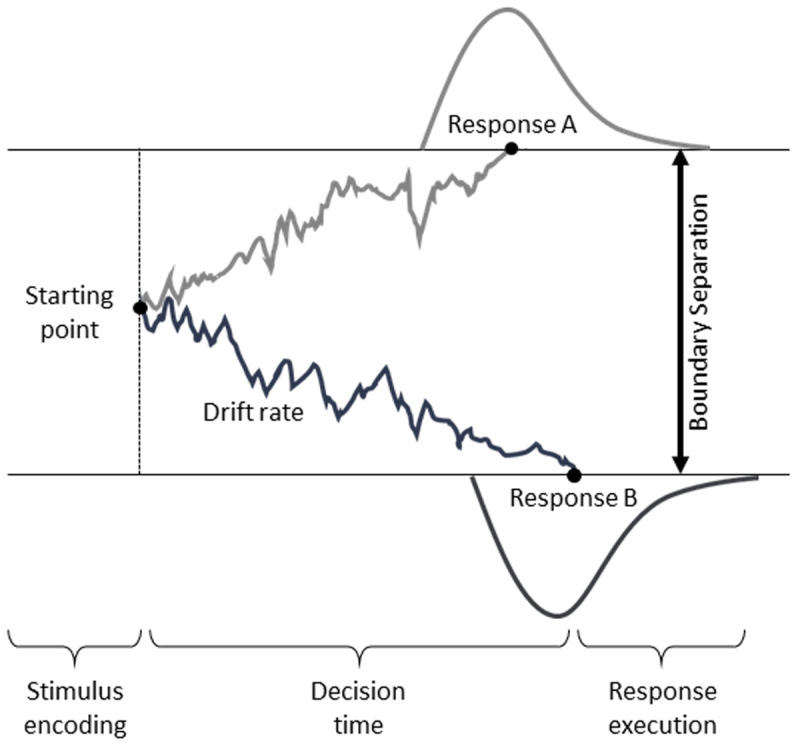
Illustration of the Drift-Diffusion Model. See main text for further information. From “Mechanisms of processing speed training and transfer effects across the adult lifespan: protocol of a multi-site cognitive training study,” by von Bastian, C. C., Reinhartz, A., Udale, R. C., Grégoire, S., Essounni, M., Belleville, S., & Strobach, T. (2022). Mechanisms of processing speed training and transfer effects across the adult lifespan: Protocol of a multi-site cognitive training study. BMC Psychology, 10, Article 168. https://doi.org/10.1186/s40359-022-00877-7. Copyright 2022 CC BY 4.0.

Cognitive models can provide more insight into response strategies and training-related mechanisms underlying changes in behavioral performance. A widely used cognitive computational model applicable to speeded decision tasks is the drift-diffusion model. The model is fit to the RT distribution and error rates simultaneously, thereby accounting for the speed-accuracy trade-off (Stafford et al., 2020), to estimate interpretable decision parameters (Ratcliff, 1978; Ratcliff & McKoon, 2008). Figure 1 illustrates the components of the decision-making process in Ratcliff’s (1978) diffusion model. The model distinguishes the rate of a noisy evidence accumulation process from response strategies and processes unrelated to the decision itself, such as stimulus encoding and motor response (Ratcliff, 1978; Ratcliff & McKoon, 2008).

In Ratcliff’s (1978) diffusion model, the evidence accumulation begins after the encoding of a presented stimulus, for example, a familiar or an unfamiliar face. The starting point of the evidence accumulation process reflects possible *a priori* biases towards either response option. In Figure 1, the starting point is located in the middle between the two response options reflecting no bias, while a bias would be indicated by a starting point shifted to one of the response options. The slope of the evidence accumulation process indicates how efficiently stimulus information is processed and is quantified as the drift rate (*v*). Reaching the threshold for either response option (e.g., that the presented face is familiar or unfamiliar) indicates that sufficient evidence has been accumulated to execute the decision. The distance between the two response thresholds is the boundary separation (*a*). The boundary separation reflects response caution and, thus, is an indicator for the speed-accuracy trade-off. The boundary separation can be wide, indicating high response caution, thus favoring accuracy and accumulating more evidence prior to a decision, or narrow, indicating low response caution, favoring speed and accumulating less evidence prior to a decision. Ultimately, when a decision has been made, a corresponding response has to be executed by, for example, pressing a response key. The non-decision time (*t_0_*) captures the time for any other processes not directly related to the decision itself, for example, encoding the stimulus and executing the motor response.

### Training-Related Changes in Decision Parameters

Previous studies have shown that repeatedly performing a task can affect evidence accumulation, response caution and, occasionally, non-decision processes. Several studies found that, when repeatedly performing the same speeded task for up to six sessions, the speed of evidence accumulation increases (Dutilh et al., 2011; Dutilh et al., 2009; Liu & Watanabe, 2012; Petrov et al., 2011; Ratcliff et al., 2006; van Ravenzwaaij et al., 2014; Zhang & Rowe, 2014). This indicates that training may improve the efficiency of information processing and suggests that increased drift rates are one factor underlying the decrease in response times that is consistently observed when repeatedly performing a task (Heathcote et al., 2000; Logan, 1992; Newell & Rosenbloom, 1981). Another factor that contributes to the decrease in response times is a shift in response strategy after repeated task practice. While drift rates increase during brief periods of training, the boundary separation indeed often decreases (Dutilh et al., 2011; Dutilh et al., 2009; Liu & Watanabe, 2012; Petrov et al., 2011; Ratcliff et al., 2006; Zhang & Rowe, 2014). Therefore, training can elicit a shift in response caution leading people to focus more on speed rather than accuracy. Training-related changes elicited in non-decision time are less consistent. Some studies have shown a decrease in this decision parameter (Dutilh et al., 2011; Dutilh et al., 2009; Petrov et al., 2011), while other studies suggest that repeatedly performing a task does not substantially affect perceptual and motor processing (Pashler & Baylis, 1991; Strobach et al., 2013).

Training-related improvements in evidence accumulation combined with an increased focus on speed, as opposed to accuracy, can explain why decreased RTs after training are still often accompanied by increased accuracy (Liu & Watanabe, 2012; Petrov et al., 2011; Ratcliff et al., 2006; Zhang & Rowe, 2014). More efficient information processing has been argued to counteract the decrease in response caution, thus, allowing for error rates to remain low. However, whether error rates decrease also depends on the type of feedback provided during task performance. For example, Dutilh and colleagues (2009) provided accuracy-related feedback to one half of their participants and speed-related feedback to the other half. The group focusing on accuracy made less errors already at the start of training and mainly improved in their RTs. The group focusing on speed showed the opposite pattern, with faster responses already at the start of training and mainly improving in accuracy. This demonstrates that the type of feedback provided when repeatedly performing a task influences not only the initial task performance but also the pattern of change during training. However, the type of feedback provided during training of speeded decision tasks has varied greatly in previous studies. While some studies have experimentally varied accuracy versus speed-related feedback (Dutilh et al., 2009; Zhang & Rowe, 2014), others have provided feedback either only for slow responses (Liu & Watanabe, 2012, van Ravenzwaaij et al., 2014), for slow, fast, and incorrect responses (Dutilh et al., 2011; Ratcliff et al., 2006) or instead given bonus points for correct responses (Petrov et al., 2011). Therefore, it remains unclear how training affects drift-diffusion parameters when consistent feedback is given on all responses throughout training.

Moreover, previous studies investigating changes during training used only a single training task during their training. This might be problematic, because it remains unclear whether changes observed are task-specific or task-general (Shipstead et al., 2012; von Bastian et al., 2019). In addition, these studies mainly used only one of two types of perceptual discrimination tasks, that is, either dot-motion detection tasks (Liu & Watanabe, 2012; Petrov et al., 2011; van Ravenzwaaij et al., 2014; Zhang & Rowe, 2014) or verbal discrimination tasks (Dutilh et al., 2011; Dutilh et al., 2009; Ratcliff et al., 2006). The limited variability in speeded decision tasks limits the generalizability of previously reported training-related changes in decision parameters and raises the question whether the same effects emerge in a wider variety of decision tasks. A recent paper by Schmiedek and colleagues (2022) addressed this concern by analyzing the difference in performance on three different choice-RT tasks before and after an extensive 100-session training intervention. The results showed pre- to post-training increases in drift rate and decreases in boundary separation and non-decision time across these different tasks. However, Schmiedek et al. (2022) did not report on changes occurring during training.

Further limitations of the previous studies investigating changes during training concern the duration of training and the number of participants included in the studies. While cognitive training interventions typically consist of 10 to 20 sessions performed over several weeks (von Bastian, Belleville, et al., 2022) and even up to 100 sessions (e.g., Schmiedek et al., 2010, 2022), the training-related changes in decision parameters have only been investigated for six training sessions or less (Dutilh et al., 2011; Dutilh et al., 2009; Liu & Watanabe, 2012; Petrov et al., 2011; Ratcliff et al., 2006; van Ravenzwaaij et al., 2014; Zhang & Rowe, 2014). Also, in most previous studies, sample sizes were small, ranging from 4 to 14 participants (Dutilh et al., 2011; Dutilh et al., 2009; Liu & Watanabe, 2012; Zhang & Rowe, 2014), increasing the risk of false-positive findings (Button et al., 2013). Changes in the drift-diffusion model parameters studied with accuracy-versus-speed group manipulations need to be interpreted with caution when, for example, groups consisted of only two participants each (Dutilh et al., 2009). Taken together, although the previous studies provide tentative evidence supporting changes in drift-diffusion parameters when repeatedly performing a single task, it remains unclear how these decision-making components change during an extensive, prolonged training regime with varied decision tasks and performance-based, trial-by-trial feedback.

### Present Study

In the present study, we fit the drift-diffusion model to existing data of a previously published, multi-session processing speed training intervention (von Bastian & Oberauer, 2013). During this training intervention, 30 participants performed 20 training sessions over the course of four weeks, completing three different choice-RT tasks each session: a face-matching, a pattern-matching, and a digit-matching task. These tasks require evidence accumulation regarding whether two stimuli that are presented simultaneously are the same. Therefore, in our application, the diffusion model does not describe the processing of a single stimulus as in the original model by Ratcliff (1978). However, this model instead describes the decision-making process on a more general level, which is similar to previous studies that used tasks with similar (e.g., dot-motion detection tasks; Liu & Watanabe, 2012; Petrov et al., 2011; van Ravenzwaaij et al., 2014; Zhang & Rowe, 2014; verbal discrimination tasks; Dutilh et al., 2011; Dutilh et al., 2009; Ratcliff et al., 2006; and other choice-RT tasks; Schmiedek et al., 2022) or even higher complexity, such as judging the correctness of mathematical equations (Lerche et al., 2020).

The use of three different tasks enables the comparison of training-related changes in different perceptual contexts and task difficulties. Each task consisted of 500 trials per session, thereby providing 30,000 trials per participant over the course of training and, thus, a considerably higher trial count compared to previous studies. Unlike previous studies, no specific speed- or accuracy-related manipulations were applied in the present study; participants were instructed to perform both accurately and quickly during all trials when deciding whether the two presented stimuli portrayed the same face, same pattern, or same series of digits. After each trial, feedback on the correctness of the participant’s response was provided. The presentation time of the stimuli was adjusted adaptively based on the participant’s performance. Therefore, by providing variability in tasks, consistent feedback, and adaptive training, this study’s training regime made use of components that are crucial to successful training (Schmidt & Bjork, 1992) and offers an ideal dataset to investigate the changes in training-related mechanisms during an extensive processing speed training.

With this study, we examined whether decision parameters estimated by the drift-diffusion model show similar patterns during extensive training as previously found during brief training periods. Therefore, throughout the course of the 20 training sessions, we expected participants to increase in their efficiency of evidence accumulation, that is, their drift rate (Hypothesis 1). Furthermore, we expected a decrease in boundary separation over time, thereby indicating that participants decrease their response caution and increasingly emphasize speed over accuracy as their training progresses (Hypothesis 2). We did not expect to see such training-related changes in non-decision time and assumed perceptual and motor processing to remain unaffected by extensive training (Hypothesis 3). Additionally, we aimed to understand whether training-related changes differed for the different choice-RT tasks included (speeded face-, digit-, and pattern-matching tasks). Therefore, we tested our hypotheses using the type of task as a predictive factor in our analyses.

## Methods

The present methods section provides information relevant to the processing speed training group of a previously published training study. For a full overview of the study design and results beyond this training group, the reader is referred to von Bastian and Oberauer (2013).

### Participants

Participants of von Bastian and Oberauer (2013) were recruited for a “cognitive training study” at the University of Zurich via the university’s subject pool and using flyers. They were randomly assigned to 1 of 4 training groups practicing either different facets of working memory or practicing processing speed as an active control training compared to the working memory training groups. In the current study, we focus only on the active control group, which included 30 young adults (21 female, 9 male, *M* age = 23.77 years, *SD* = 4.73 years). All participants gave informed written consent for participation prior to testing. After completion of the study, participants received monetary compensation (CHF 150) or course credits.

### Design

Study participation consisted of 4 weeks of training, in which participants were asked to perform 20 training sessions. Each session took about 30–40 minutes. Participants performed their training on their home computer via Tatool (von Bastian, Locher, & Ruflin, 2013). Therefore, participants could decide themselves when they preferred to perform their training. At the end of each training session, training data were automatically uploaded to a web server. Whereas self-administering training at home increases ecological validity, it can also lead to loss of experimental control. Several measures were taken to assure active and conscious execution of the training program, such as signing a participation agreement that training completion will be taken seriously, monitoring training data to detect irregularities, and maintaining regular email contact with the participants as well as providing technical support when needed.

### Materials

**Figure 2:**
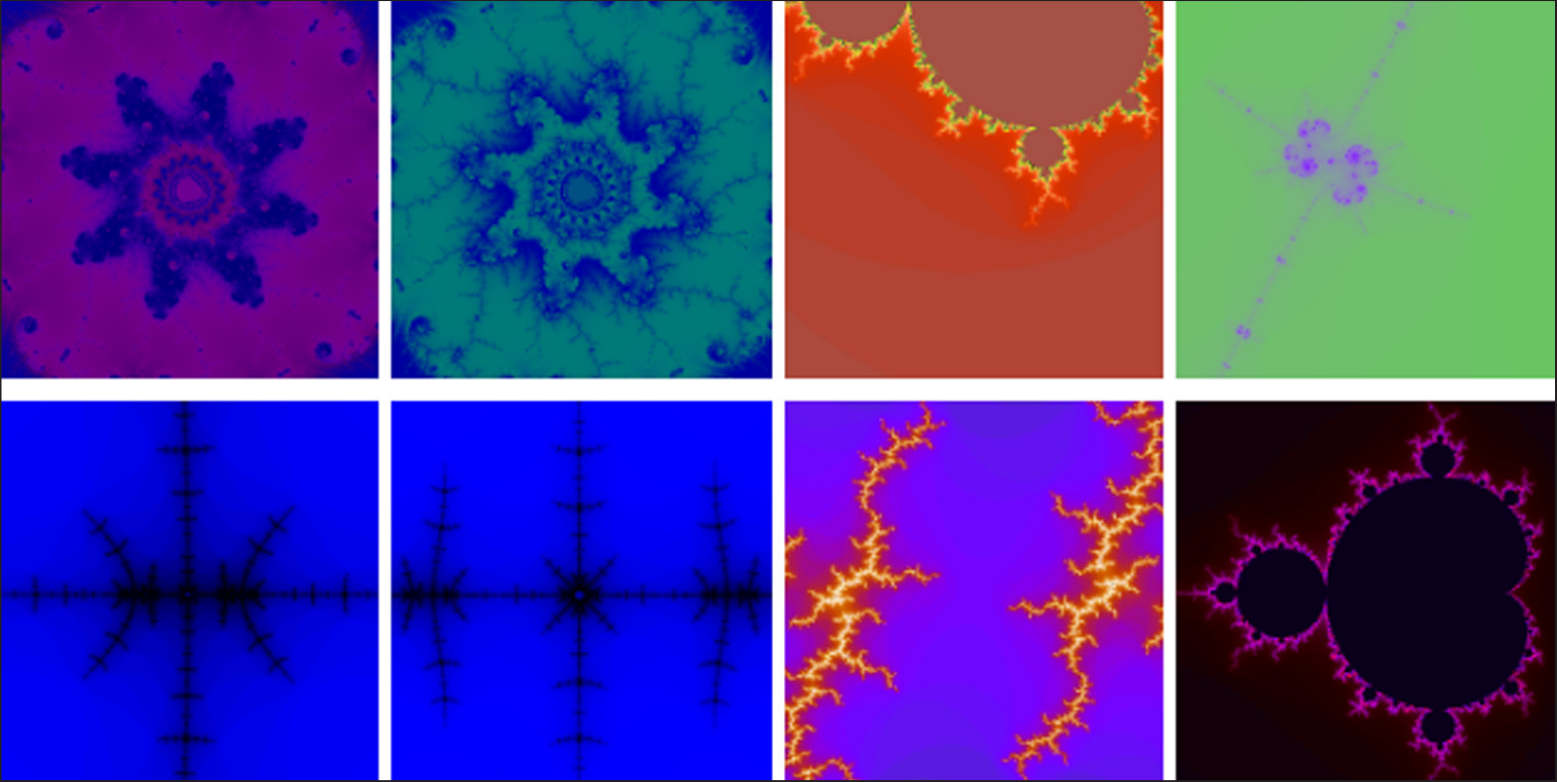
Examples of Fractal Patterns in the Pattern-Matching Task.

Each training session consisted of three tasks with 500 trials each, in the following order: face-matching, digit-matching and pattern-matching, with breaks offered between tasks. During these tasks, participants were presented with a pair of stimuli for which they had to decide whether the pair showed the same person (face matching), the same six-digit number (digit matching), or the same fractal pattern (pattern matching). The face stimuli were 12 pictures each from 84 people (42 female, 42 male) with different facial expressions or accessories, taken from the AR face database (Martinez & Benavente, 1998). The digit stimuli were six-digit numbers presented in the same font. The pattern stimuli were 305 computer-generated squares of fractal patterns of various bright colors and shapes (see Figure 2 for some examples).

During all tasks, participants were asked to respond as quickly and as accurately as possible to the stimulus pair by pressing the “A” key for a matching stimulus pair, and the “L” key for a non-matching pair. After every trial, participants received feedback about the correctness of their response with a green check, indicating they correctly identified the stimulus pair as either matching or non-matching and a red cross indicating an incorrect response. The trial type (matching vs. non-matching), the matching stimulus pairs, and the combinations of non-matching pairs were selected at random. The intertrial interval was 100 ms. The stimuli were displayed until the participant’s response was registered or until the maximum stimulus presentation was exceeded. All participants started training with a maximum stimulus presentation duration of 5000 ms. During training, this maximum stimulus presentation was reduced adaptively based on the participant’s performance in the preceding 40% of trials to increase the difficulty of the tasks and to motivate participants to continuously focus on both speed and accuracy (see von Bastian & Oberauer, 2013, for more details on the adaptive level algorithm). The increase in difficulty was minor, however, with the maximum response time set to the 99th percentile of the RTs of those trials completed after the last reduction.

## Results

### Data Preparation and Parameter Estimation

#### Missing Data

Training data were partially missing for five participants. For one participant, the entire 16th session was not recorded. For another participant only about half of the trials of the face-matching task were recorded during the first session, and during four other sessions their pattern-matching data were incomplete, which was the final task in each session. Three other participants also had incomplete pattern-matching data, one of them for two sessions, the others for one session each. As in the previously published paper, the fully missing session was excluded from further analyses (von Bastian & Oberauer, 2013). Training sessions with partially missing data were subjected to the same data treatment procedures as fully recorded training sessions.

#### Data Treatment

Before fitting the drift-diffusion model to the training data, a number of factors were considered first. Based on the findings from a series of simulation studies by Lerche et al. (2017), when using Kolmogorov-Smirnov as optimization criterion to estimate a three-parameter model a minimum trial number of *N* = 125 is recommended to obtain robust estimates with at least low precision (see also Voss et al., 2013). This minimum *N* is recommended for data without fast and slow RT contaminants and with a minimum of 4% of each response type that, in our case, are correct and incorrect responses. Slow contaminants in particular hamper the estimation, especially of the boundary separation which would otherwise require at least 555 trials per participant, task, and session (Lerche et al., 2017).

To remove fast and slow contaminants, we excluded all trials with RTs below 250 ms and those with RTs that were 2.5 median absolute deviations (MAD) above the median (Leys et al., 2013). We also explored other frequently used cut-offs (e.g., 2 SDs) but decided to use the more conservative 2.5 MADs to remove a larger number of slower contaminants. RT trimming was performed separately per task, participant, session, and response type (correct/incorrect). Subsequently, we excluded entire training sessions if they contained less than the recommended 125 trials post-RT-trimming. We then checked that each session dataset contained a minimum of 4% trials for each response type. For all tasks, all recorded training sessions contained more than 4% correct responses. However, there were a number of sessions with less than 4% incorrect responses which were excluded. Finally, participants with less than half of the training sessions remaining post-trimming (<10 training sessions) were excluded from all further analyses.

Table 1 provides an overview of the included numbers of trials, sessions, and participants. The total number of removed trials ranged from 4.91% for face matching (remaining participants *n* = 30), 12.96% for digit matching (remaining *n* = 29) and up to 38.75% for pattern matching (remaining *n* = 25), reflecting variations in task difficulty. We also considered removing trials after errors because people adjust their response behaviors in various ways post-error (for an overview, see Danielmeier & Ullsperger, 2011); however, trimming of post-error trials prior to the listed data treatment steps would have increased the number of removed trials to 17.84% for face matching, 23.01% for digit matching and even 44.67% for pattern matching. Since the inclusion of post-error trials did not qualitatively alter the results of the analyses, we decided to include post-error trials to maximize the number of trials for better estimation precision.

**Table 1:** Overview of Trial, Session, and Participant Count per Task.


	TASK		

	FACES	DIGITS	PATTERNS

**Data Recorded (N)**			

Trials	299,260	299,000	296,799

Sessions	599	598	598

Participants	30	30	30

**Data Treatment Steps (% Remaining Trials)**			

1. RT (<250 ms; >2.5 MAD)	95.09%	95.48%	94.84%

2. Sessions (<125 trials)	–	–	94.75%

3. Sessions (<4% errors)	–	88.32%	64.61%

4. Participants (<10 sessions)	–	87.04%	61.25%

**Data Post-Treatment (N)**			

Trials	284,564	260,256	181,783

Sessions	599	545	385

Participants	30	29	25


*Note*. This table summarizes the number of trials, sessions, and participants in each task before and after data treatment steps.

#### Model Estimation and Fit

**Figure 3:**
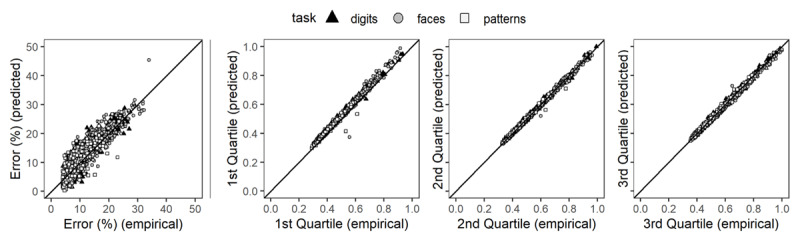
Graphical Illustration of Model Fit as a Function of Errors and Reaction Time Quartiles.

We estimated the drift rate (*v*), boundary separation (*a*), and non-decision time (*t_0_*) separately per task. For each task, parameters were estimated for participants and training sessions (accounting for the dependency between sessions using the “depends” fast-dm function) with a fixed starting point of 0.5 between the upper (1, correct response) and lower (0, incorrect response) threshold using the open-source fast-dm-30 software applying Kolmogorov-Smirnov as optimization criterion (Voss & Voss, 2007, 2008; Voss et al., 2015). Subsequently, using the fast-dm construct-samples tool (Voss & Voss, 2007, 2008; Voss et al., 2015), data were simulated for each task, participant, and the corresponding sessions separately, with the same number of trials as in the empirical session data. As illustrated in Figure 3, while only slightly overestimating RTs in the first quartile, the model fits the data well. Identical patterns of model fit were found for all tasks irrespective of stimuli and differences in the number of available data points post-trimming.

### Analysis of Training- and Task-Related Effects

Linear Mixed-Effects (LME) models were used to analyze the training data with the lmer function from the lme4 package (Bates et al., 2015) in R (version 4.2.1; R Core Team, 2022). The models were specified with the dependent variable (mean RT to correct responses, error rate, drift rate, boundary separation, or non-decision time) predicted by training session (1 to 20) and training task (face, digit, and pattern matching), with a random effect of participant on the intercept.^1^ Type III ANOVA test statistics were estimated for the LME models using the lmerTest package (Kuznetsova et al., 2017). Satterthwaite’s degrees of freedom were used to estimate *p*-values for the effects of session, task, and their interaction.

#### Reaction Times and Error Rates

**Figure 4:**
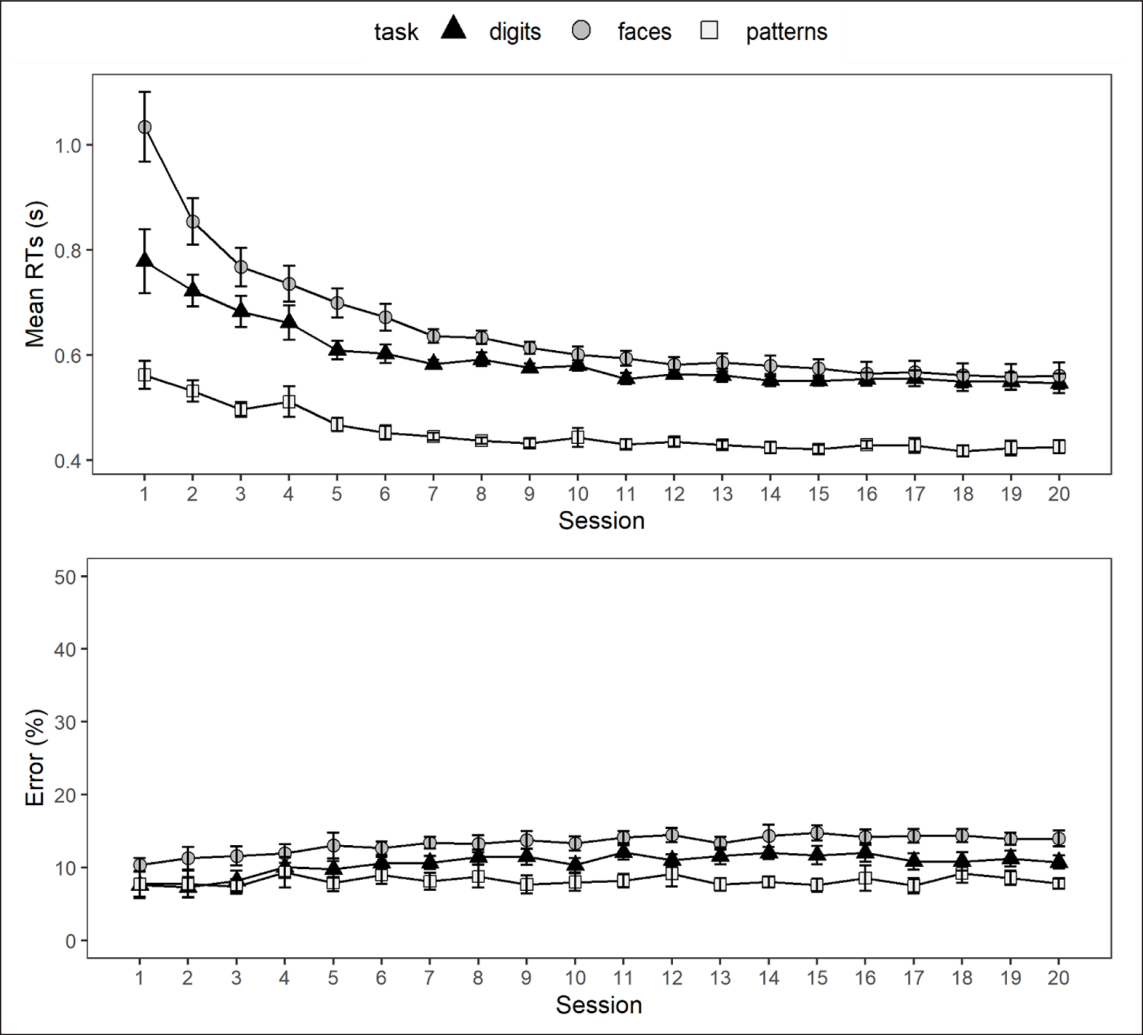
Training Effects on Mean Reaction Times (RTs) and Error Rates in the Individual Matching Tasks. RTs are to correct responses only. The error bars denote approximated 95% confidence intervals for within-subjects comparisons calculated according to Cousineau (2005) and Morey (2008). Note that there are differences in the number of available data points per session and task due to the reported data treatment.

Figure 4 illustrates performance in the training tasks over the course of 20 training sessions. For mean RT to correct responses, we found a significant main effect of session, *F*(19, 1440) = 72.49, *p* < .001, η_p_^2^ = 0.49, and of task, *F*(2, 1442) = 987.77, *p* < .001, η_p_^2^ = 0.58. Moreover, the interaction between session and task was significant, *F*(38, 1440) = 10.01, *p* < .001, η_p_^2^ = 0.21. Similarly, for error rates, the effect of session was also significant, *F*(19, 1440) = 5.80, *p* < .001, η_p_^2^ = 0.07, as was the effect of task, *F*(2, 1443) = 345.57, *p* < .001, η_p_^2^ = 0.32. However, the interaction between session and task was not significant, *F*(38, 1440) = 1.05, *p* = .391, η_p_^2^ = 0.03. For better comparability to the original analysis reported in von Bastian and Oberauer (2013), we ran the same set of analyses but including sessions with less than 4% error rates. The patterns of results were the same as for the reduced data set used in the present study, except that the interaction between session and task was then also a significant predictor of error rates, *F*(38, 1585) = 1.43, *p* = .045, η_p_^2^ = 0.03.

#### Diffusion-Model Parameters

**Figure 5:**
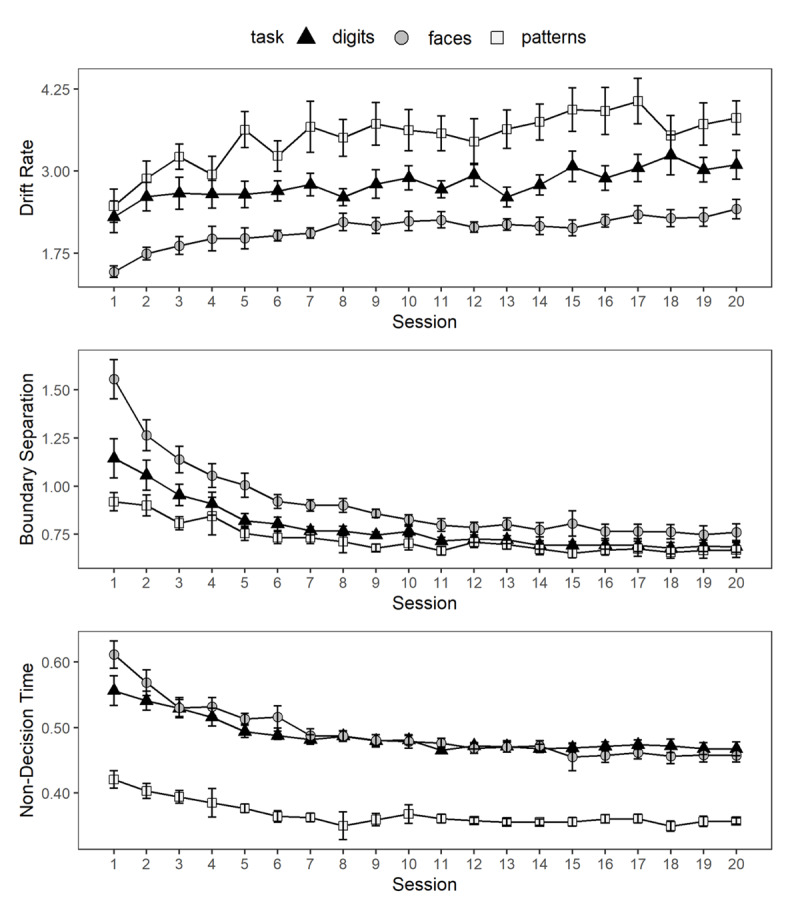
Training Effects on Diffusion-Model Parameters in the Individual Matching Tasks. The error bars denote approximated 95% confidence intervals for within-subjects comparisons calculated according to Cousineau (2005) and Morey (2008). Note that there are differences in the number of available data points per session and task due to the reported data treatment.

Figure 5 illustrates the changes in drift rate, boundary separation, and non-decision time over the course of the 20 training sessions. Drift rate increased over the course of the sessions, *F*(19, 1440) = 12.75, *p* < .001, η_p_^2^ = 0.14, and differed per task, *F*(2, 1445) = 826.48, *p* < .001, η_p_^2^ = 0.53. Also, the interaction of task and session was significant, *F*(38, 1440) = 1.78, *p* = .003, η_p_^2^ = 0.04. In contrast to the drift rate, boundary separation decreased over the course of the sessions, *F*(19, 1440) = 82.63, *p* < .001, η_p_^2^ = 0.52. The effect of task on boundary separation was significant, *F*(2, 1443) = 300.17, *p* < .001, η_p_^2^ = 0.29, as well as the interaction between task and session, *F*(38, 1440) = 6.81, *p* < .001, η_p_^2^ = 0.15. Non-decision time also decreased as an effect of session, *F*(19, 1440) = 32.97, *p* < .001, η_p_^2^ = 0.30, and differed per task, *F*(2, 1441) = 1410.51, *p* < .001, η_p_^2^ = 0.66. Session and task also interacted with regards to non-decision time, *F*(38, 1440) = 3.73, *p* < .001, η_p_^2^ = 0.09. We ran the same estimation procedures while including the otherwise excluded sessions with less than 4% error rates, which also fit the data well. The patterns of results from these subsequent analyses were the same as for the estimated parameters using the reduced data set, except that the interaction between session and task was no longer a significant predictor of drift rate, *F*(38, 1703) = 1.23, *p* = .158.

In summary, drift rates increased with training, while boundary separation and non-decision time decreased. These changes over the course of training were found across the individual participants, as shown in Figures A1-3 in the Appendix. Drift rates were highest for pattern-matching, followed by digit-matching and face-matching. The reverse pattern emerged for boundary separation which was highest in the face-matching task and lowest in pattern-matching, although this difference was most pronounced at the beginning of training. Non-decision time remained lowest for pattern-matching throughout all training sessions.

#### Model Selection

Previous research has shown that fitting more than necessary drift-diffusion model parameters can lead to trade-offs between these unconstrained parameters and, thereby, mimic experimental effects (Heathcote et al., 2015). Therefore, we systematically compared models differing in parameterization constraints. Specifically, we fixed either the drift rate, boundary separation, non-decision time, or a combination of these parameters to the median of all session means as estimated by the three-parameter model. Model comparisons confirmed that the three-parameter model fit our data best. The model fit visibly decreased when fixing one or two of the three parameters otherwise varying freely. The decrease in model fit was most pronounced when fixing the non-decision time. Fixing the drift rate or boundary separation also impacted the model fit negatively, but to a lesser extent. Critically, analyses performed on these lesser-fitting models led to the same results, except that the interaction between session and task no longer significantly predicted drift rate when fixing non-decision time (either alone or in combination with boundary separation). An overview of the model comparisons as well as the model fit of the three-parameter model including sessions with less than 4% error trials, of which the results are reported in the previous section, is available at https://osf.io/kz67v.

## Discussion

The current study investigated the mechanisms underlying training-related changes in processing speed by examining changes in the components of RT distributions estimated with the drift-diffusion model. We analyzed existing data of an extensive multi-session training intervention (von Bastian & Oberauer, 2013) to disentangle changes in drift rate, boundary separation, and non-decision time during training of three different speeded choice-RT tasks administered with trial-by-trial feedback. Our results showed that these decision-making components changed over the course of 20 training sessions with drift rates increasing in all three tasks. Boundary separation and non-decision time decreased, especially at the beginning of training. Overall, the same patterns were observed across the three training tasks with changes in boundary separation and non-decision time being most pronounced during initial training periods, whereas the rate of evidence accumulation continued to improve until later training sessions. The use of multiple tasks during an extensive amount of training sessions strengthens the implications of our study and allows for conclusions about training-related changes with regards to different task stimuli beyond their RT and error effects in a cognitive training context.

Training-related increases in drift rates confirm our hypothesis that processing speed training can improve the efficiency of evidence accumulation. This finding is consistent with previous studies with up to six training sessions that reported increased efficiency of evidence accumulation during training (Dutilh et al., 2011; Dutilh et al., 2009; Liu & Watanabe, 2012; Petrov et al., 2011; Ratcliff et al., 2006; van Ravenzwaaij et al., 2014; Zhang & Rowe, 2014). Our results from a more extensive, 20-sessions training regime show that this improvement in evidence accumulation rate extends beyond a few initial sessions. In contrast, the decreases in boundary separation seem limited to initial training sessions. These findings are in line with previous research (Dutilh et al., 2011; Dutilh et al., 2009; Liu & Watanabe, 2012; Petrov et al., 2011; Ratcliff et al., 2006; Zhang & Rowe, 2014) and our expectation that boundary separation decreases with training, indicating that participants decrease their response caution and increasingly emphasize speed over accuracy as their training progresses. Moreover, we found that this adjustment towards favoring speed occurs in the early stages of training.

Contrary to our hypothesis that perceptual and motor processes remain unaffected by extensive training, we found a training-related decrease in non-decision time. This indicates that the drift-diffusion parameters estimated from our data were sensitive to detect adjustments in non-decision processes, such as stimulus encoding and motor responses, as observed in some studies before (Dutilh et al., 2011; Dutilh et al., 2009; Petrov et al., 2011). Our three-parameter diffusion model was sensitive to detect differences in non-decision processes. Moreover, non-decision time as well as boundary separation and drift rate were crucial components that, when constrained to be the same across sessions, drastically reduced model fit. Similar to the changes in boundary separation, the decrease in non-decision time was most pronounced during the initial training sessions and then remained stable throughout the rest of training. Overall, training-related changes in drift-diffusion parameters were similar across the different training tasks, with relatively negligible variations between the tasks.

The changes in drift-diffusion parameters shed light on the mechanisms underpinning the training-related changes typically observed in mean RTs. In general, participants sped up during training, as is to be expected when repeatedly performing the same tasks (Heathcote et al., 2000; Logan, 1992; Newell & Rosenbloom, 1981). Shorter RTs as a result of processing speed training can, thus, be explained by an increased efficiency of evidence accumulation as well as a decrease in response caution and non-decision processes. Typically – as in the present study – the training-related decrease in mean RTs is most pronounced during the first few training sessions and then seems to slow down. Our findings show that the steep decrease in the first few training sessions is driven by changes in all three major drift-diffusion parameters (drift rate, boundary separation, and non-decision time). The three main parameters of the drift-diffusion model are simultaneously changing during the initial part of training, all in manners that reduce RTs, that is, more efficient evidence accumulation, narrower boundary separation, and shorter non-decision time.

In the current study, the decrease in RTs was accompanied by an increase in error rates (that is, decreased accuracy), suggesting a speed-accuracy trade-off. As training progresses participants increasingly favored speed over accuracy, as indicated by their decrease in boundary separation. Previous studies reported increases in accuracy even when the boundary separation decreased (and the rate of evidence accumulation increased; Dutilh et al., 2009; Liu & Watanabe, 2012; Petrov et al., 2011; Ratcliff et al., 2006; Zhang & Rowe, 2014). However, these former studies applied different types of feedback regarding either the speed or accuracy of task performance and were performed in laboratory settings. Therefore, they did not require participants to incorporate an extensive training regime into their day-to-day life at home which possibly motivated participants in the current study to focus more on speed than accuracy. Furthermore, in our study, participants already started their training with low error rates and were provided feedback on the accuracy of their responses throughout training. Therefore, participants will have received mainly positive feedback on their correctness, and therefore, may have focused more on improving their RTs, which led to a decrease in accuracy despite improved evidence accumulation rates. In another study providing only accuracy-related feedback as opposed to speed-related feedback, participants also started training with low error rates and improved their RTs but not their accuracy (Dutilh et al., 2009).

While the overall pattern of results was highly similar across all three tasks, our results suggest that the pattern-matching task was easiest to perform, possibly followed by the digit-matching and the face-matching task. The face and digit stimuli as opposed to the pattern stimuli elicited the most pronounced training-related changes throughout the majority of observed measures and drift-diffusion parameters. Mean RTs and error rates were lowest for this task throughout the training regime and this difference in task difficulty was also visible in the drift-diffusion parameters. Drift rates were highest during pattern-matching, followed by digit-matching and then lowest during face-matching across all training sessions. In comparison, boundary separation and, especially, non-decision time was considerably lower for pattern stimuli than for face and digit stimuli. The difference in non-decision time throughout training might indicate that the pattern stimuli differed with regards to their non-decision processes, presumably their perceptual encoding (since motor processes were identical between the tasks).

The observed patterns of change in drift-diffusion parameters across tasks may be explained by Chein and Schneider’s (2012) triarchic theory of learning. The early changes in non-decision time and boundary separation may represent the formation stage, in which processes of establishing new behavioral routines are established to learn how to approach and execute the task. In this stage, learning relies strongly on the metacognitive system and the use of strategies. In the next stage of learning, the metacognitive system is less involved and, instead, the cognitive control network is engaged to efficiently execute the new behavioral routines. Possibly, the more prolonged improvements in evidence accumulation reflect this second stage of learning. The final stage in Chein and Schneider’s (2012) model is the automatic execution stage, during which the behavioral routine becomes increasingly automatized.

### Limitations and Outlook

The use of multiple tasks during an extensive amount of training sessions strengthens the implications of the current study and allows for conclusions about training-related changes with regards to different task stimuli beyond their initial training effects. However, direct comparisons between the three tasks must be interpreted with caution. First, the drift-diffusion parameters were generated separately per task which might limit the comparability of the exact values of the parameters between these tasks. This limitation does not, however, hold for the RT and error data which show effects equivalent to the parameters. Therefore, we can compare the parameters between tasks at least to some degree. Second, the difficulty of the training tasks was consistent with the order of the trained tasks within each training session. Thus, it is possible that there was a confounding effect between task difficulty and task order during training: the most difficult task of face-matching was performed first, while the seemingly easiest task of pattern-matching was performed last and the digit-matching task in-between. This raises the question whether participants improved within individual training sessions which led them to perform best during the final pattern-matching task. Since the difference between tasks was present in all measures and drift-diffusion parameters, we cannot completely exclude an impact of this confound between task order and task difficulty.

Given our results, we recommend that future training studies apply an extensive training regime with at least 10 training sessions and multiple training tasks in order to disentangle the differential effects of session and task. However, we also recommend accounting for differences in task difficulty and task order during training. Furthermore, it would be of great value to investigate how training-related changes relate to transfer effects on untrained tasks of similar or different cognitive domains. Few and only recent studies have applied drift-diffusion analyses to analyze transfer effects in working memory tasks (e.g., Chen et al., 2022; Schmiedek et al., 2022). Findings from these studies suggested that pre- to post-training effects in processing speed tasks (Schmiedek et al., 2022) are similar to those observed in our study during training. To study the associations between training-related change in drift-diffusion parameters and transfer effects, a certain number of trials is required, as discussed in the results section. Further, a substantial number of participants is required, as stable correlations are obtained with a minimum of 250 participants (Schönbrodt & Perugini, 2013). The present study does not include this substantial number and, therefore, we were not able to associate the parameter changes during training with transfer measures.

Due to this study’s sample size, we were also not able to investigate individual differences during training and possible differences in training curves and effects. Overall, the stated training-related changes were found across individual participants, with potential for individual variation mainly in the drift rate (see Figures A1-3 in the Appendix). Future studies with larger statistical power are required to investigate such individual variation in drift-diffusion model parameters, ideally with Bayesian hierarchical approaches (e.g., Heathcote et al., 2019; Wiecki et al., 2013) which provide more flexibility in modeling individual differences over time.

## Conclusion

The results of the current study show that extensive training of processing speed tasks elicits changes in drift-diffusion parameters. Processing speed training improves the efficiency of evidence accumulation and this process of improving efficiency continues throughout multiple sessions of an extensive training regime. In comparison, changes in response strategy, as indicated by a decrease in response caution and emphasis on speed over accuracy, occur earlier and are limited to more initial parts of training. Non-decision processes, such as stimulus encoding and motor responses, also improve early-on during training and these improvements stabilize quickly during the first training sessions. This overall pattern of prolonged training-related changes in rate of evidence accumulation as well as early changes in response strategy and non-decision processes is detectable across different tasks with different visual stimuli. Future research could shed light into whether and how these training-related changes during processing speed ultimately affect transfer to untrained tasks of other cognitive domains or assessing transfer to everyday life functioning.

## Data Accessibility Statement

Data and analysis scripts reported in this manuscript are available at the Open Science Framework (https://osf.io/kz67v/).

## Additional File

The additional file for this article can be found as follows:

10.5334/joc.310.s1 Appendices. Figures A1 to A3.

## Footnotes

Models with additional random effects on the participant x session interaction (see Barr et al., 2013) were tested but did not converge, likely due to the complexity of the model structure not being supported by the statistical power (see Matuschek et al., 2017).
