# Supplementary material for: Mechanisms of Training-Related Change in Processing Speed: A Drift-Diffusion Model Approach
Source: J Cogn. 2023 Aug 18;6(1):46. doi: 10.5334/joc.310 (PMC10437139; doi:10.5334/joc.310)

Appendix

Figure A1: Training-Related Changes in Drift Rate per Participant. The mean drift rate of each training session averaged across tasks (face-, digit-, and pattern-matching task) is shown in an individual box per participant (numbers 1 through 30).

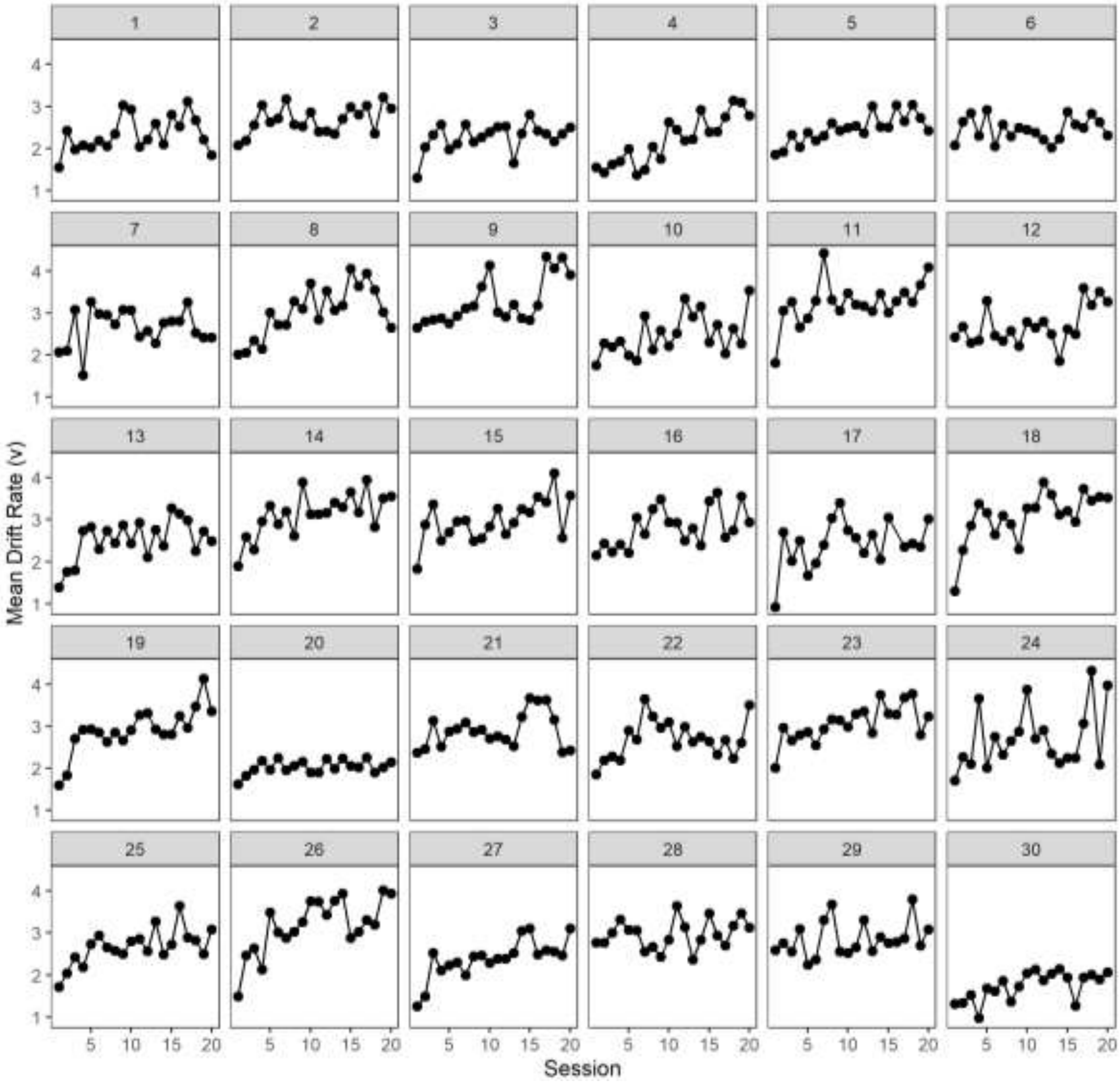

Figure A2: Training-Related Changes in Boundary Separation per Participant. The mean boundary separation of each training session averaged across tasks (face-, digit-, and pattern-matching task) is shown in an individual box per participant (numbers 1 through 30).

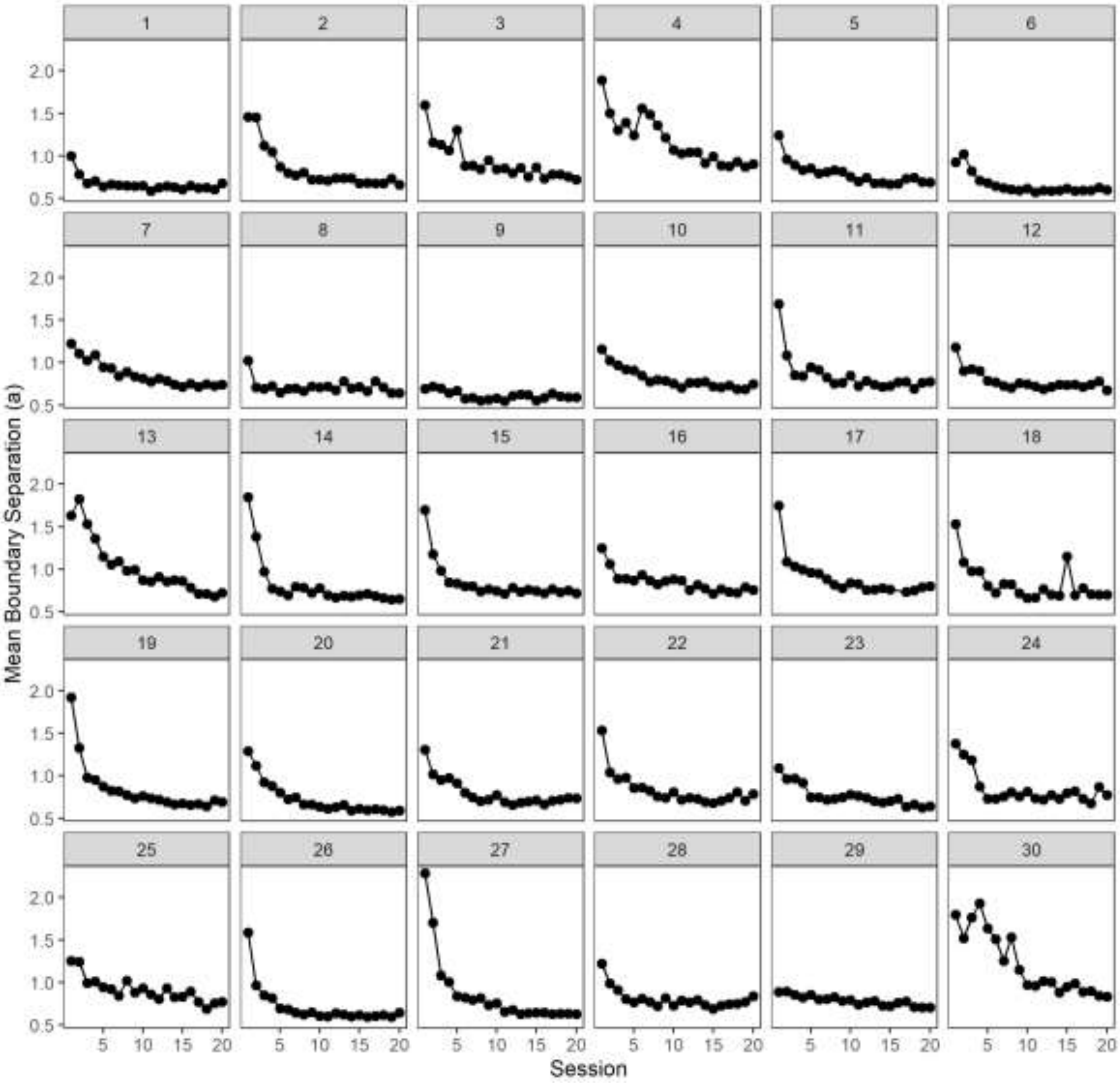

Figure A3: Training-Related Changes in Non-Decision Time per Participant. The mean non-decision time of each training session averaged across tasks (face-, digit-, and pattern-matching task) is shown in an individual box per participant (numbers 1 through 30).

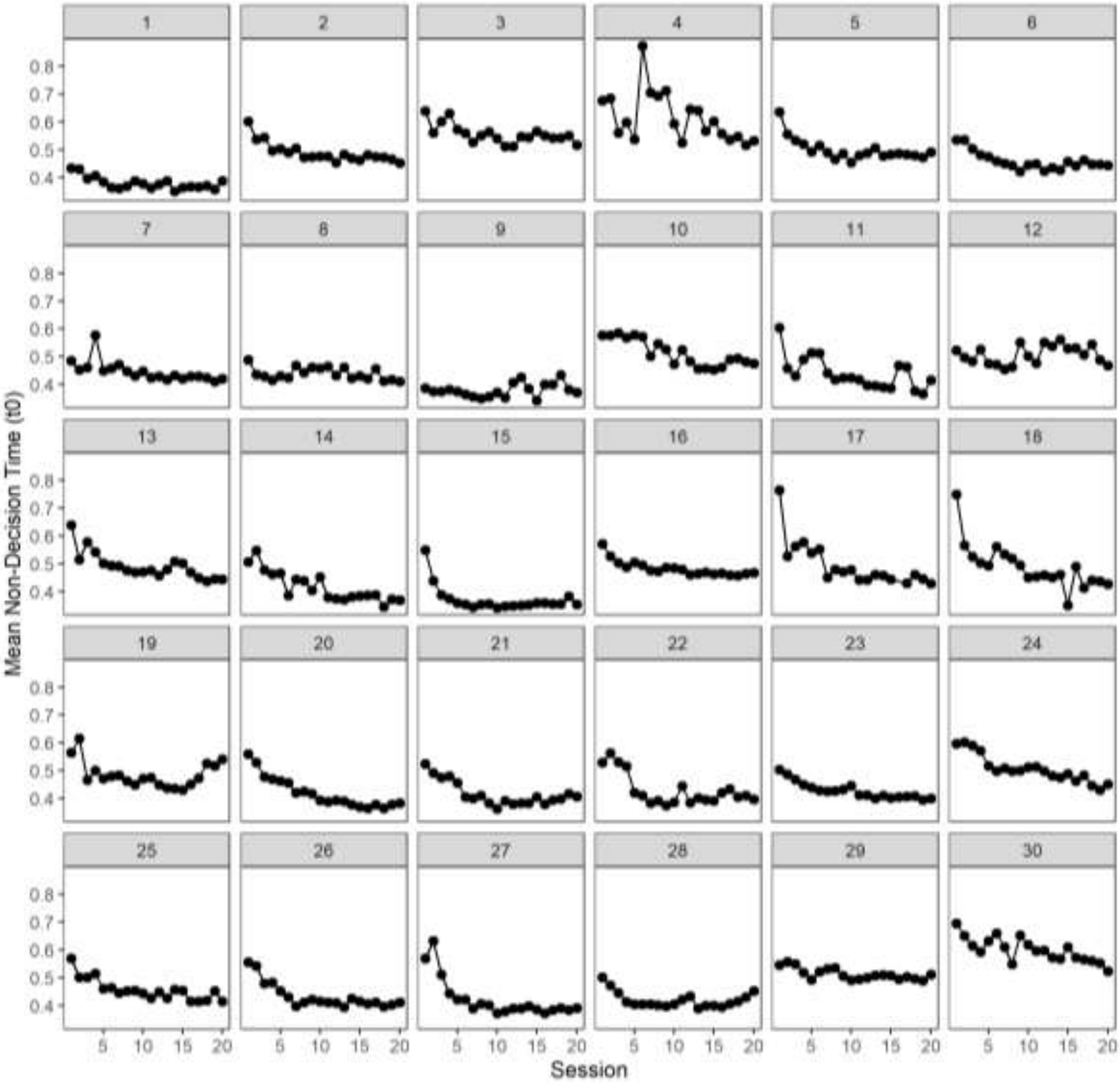

Supplement: Appendices. — Figures A1 to A3. [file joc-6-1-310-s1.pdf]
